# Blood and MRI biomarkers of mild traumatic brain injury in non-concussed collegiate football players

**DOI:** 10.1038/s41598-023-51067-3

**Published:** 2024-01-05

**Authors:** Eunhan Cho, Joshua Granger, Bailey Theall, Nathan Lemoine, Derek Calvert, Jack Marucci, Shelly Mullenix, Hollis O’Neal, Tomas Jacome, Brian A. Irving, Neil M. Johannsen, Owen Carmichael, Guillaume Spielmann

**Affiliations:** 1 School of Kinesiology, Louisiana State University, Huey P. Long Fieldhouse, Baton Rouge, LA 70803 USA; 2 LSU Athletics, LSU, Baton Rouge, LA 70803 USA; 3 Louisiana State University Health Sciences Center, Baton Rouge, LA 70803 USA; 4 Our Lady of the Lake, Baton Rouge, LA 70810 USA; 5 Pennington Biomedical Research Center, Baton Rouge, LA 70808 USA

**Keywords:** Risk factors, Diagnostic markers

## Abstract

Football has one of the highest incidence rates of mild traumatic brain injury (mTBI) among contact sports; however, the effects of repeated sub-concussive head impacts on brain structure and function remain under-studied. We assessed the association between biomarkers of mTBI and structural and functional MRI scans over an entire season among non-concussed NCAA Division I linemen and non-linemen. Concentrations of S100B, GFAP, BDNF, NFL, and NSE were assessed in 48 collegiate football players (32 linemen; 16 non-linemen) before the start of pre-season training (pre-camp), at the end of pre-season training (pre-season), and at the end of the competitive season (post-season). Changes in brain structure and function were assessed in a sub-sample of 11 linemen and 6 non-linemen using structural and functional MRI during the execution of Stroop and attention network tasks. S100B, GFAP and BDNF concentrations were increased at post-season compared to pre-camp in linemen. White matter hyperintensities increased in linemen during pre-season camp training compared to pre-camp. This study showed that the effects of repeated head impacts are detectable in the blood of elite level non-concussed collegiate football players exposed to low-moderate impacts to the heads, which correlated with some neurological outcomes without translating to clinically-relevant changes in brain anatomy or function.

## Introduction

Sports-related concussion, or mild traumatic brain injury (mTBI), is a primary concern among professional and collegiate collision sport athletes. Intracranial injuries resulting from sudden mechanical force and rapid change in velocity through acceleration and deceleration to the head characterize mTBI^1^. Among contact sport athletes, American football players appear to be at the greatest risk of developing mTBI and severe concussions due to the repetition and high intensity of in-practice and in-game accelerations/decelerations to the head^1,2^. Although the term concussion is often interchangeable with a “mild” form of TBI, historically, less attention has been placed on the deleterious effects of sub-concussive repeated head impacts on brain structure and function. This is of particular clinical relevance in sports where the repeated impacts are low to moderate force and typically would not prompt a diagnosis of mTBI. Moreover, as contact sport athletes with a history of sport-related concussions are more likely to experience recurrent concussions within sports than those with no concussion history^3^, identifying sensitive biomarkers of brain damage is crucial. This is especially true among collegiate athletes who exhibit an intensified desire for continued on-field participation and are more likely to ignore or underreport symptoms of mTBI than their professional counterparts and return to play before full recovery^3^.

While common symptoms of mTBI are well characterized, with cognitive, neuropsychological, and functional impairments routinely observed in concussed players^1,2^, similar decrements have been observed in athletes exposed to sub-concussive head impacts^4^. Whether repeated sub-concussive head impacts also increase traditional blood biomarkers of mTBI and can lead to changes in brain structure and function in non-concussed players remains unknown. Neuroimaging techniques, such as computed tomography and magnetic resonance imaging (MRI), are important research tools to investigate mTBI resulting from direct, forceful head impacts, but need further validation as clinical tools^2^. However, limited data exist regarding well-established neuroimaging markers of repeated sub-concussive impacts. Moreover, clinical characterization of functional disturbances in brain structure in response to sub-concussive head impacts remains elusive^2^.

Identifying sensitive and reliable blood biomarkers of cerebral damage induced by repeated sub-concussive head impacts would be the most ecologically relevant approach due to their minimally invasive and cost-effective nature. Several potential surrogate biomarkers for mTBI have been identified in humans^5,6^. Damage from mTBI compromises the blood–brain barrier and allows CNS-related proteins into the bloodstream through the lymphatic system. For example, S-100 calcium-binding protein B (S100B), and glial fibrillary acidic protein (GFAP), which have low concentrations in people without mTBI, can be elevated when the blood–brain barrier has increased permeability^5^. As such, increased levels of biochemical markers may represent neuroanatomical injury to the astroglia cell, the neuron cell, and the axon in the brain^5,6^. While several brain-derived proteins have been found to be elevated in the blood of contact sports athletes and TBI patients, including, brain-derived neurotrophic factor (BDNF), neurofilament light chain protein (NFL), and neuron-specific enolase (NSE)^7–11^, conflicting results between studies have limited the ability to translate those findings onto the field. Moreover, few studies have examined the changes in various blood biomarkers of brain insult with structural and functional MRI data over an entire season in non-concussed collegiate football athletes, a population at particular risk of developing impact-induced mTBI. Therefore, in this study, we aimed to examine the effect of pre-season camp and a competitive season on changes in the concentration of circulating S100B, GFAP, NSE, NFL, and BDNF in non-concussed elite-level collegiate football players, based on their position and likelihood of head impact (linemen vs. non-linemen). Furthermore, we aimed to examine the relationship between biomarkers of mTBI and structural MRI (sMRI) and functional MRI (fMRI) outcomes over the course of a competitive season.

## Results

Forty-eight male collegiate athletes (linemen: 32; non-linemen: 16) participated in this study throughout three competitive seasons (Table 1)**.** 11 linemen players and 6 non-linemen players underwent sMRI and fMRI. Linemen were taller (p < 0.0001) and heavier than non-linemen (p < 0.0001). None of the participants exhibited symptoms of, or were diagnosed with, a concussion throughout the study.

**Table 1:** Participant characteristics.

	Linemen (n = 32)	Non-linemen (n = 16)
Age (year)	19.97 ± 1.40	20.44 ± 1.55
Height (cm)	193.64 ± 2.99	186.61 ± 4.90**
Weight (kg)	136.44 ± 12.94	94.83 ± 7.28**

**Difference from non-linemen group with significant set as p < 0.0001.

### Changes in biomarkers throughout the season

**Figure 1:**
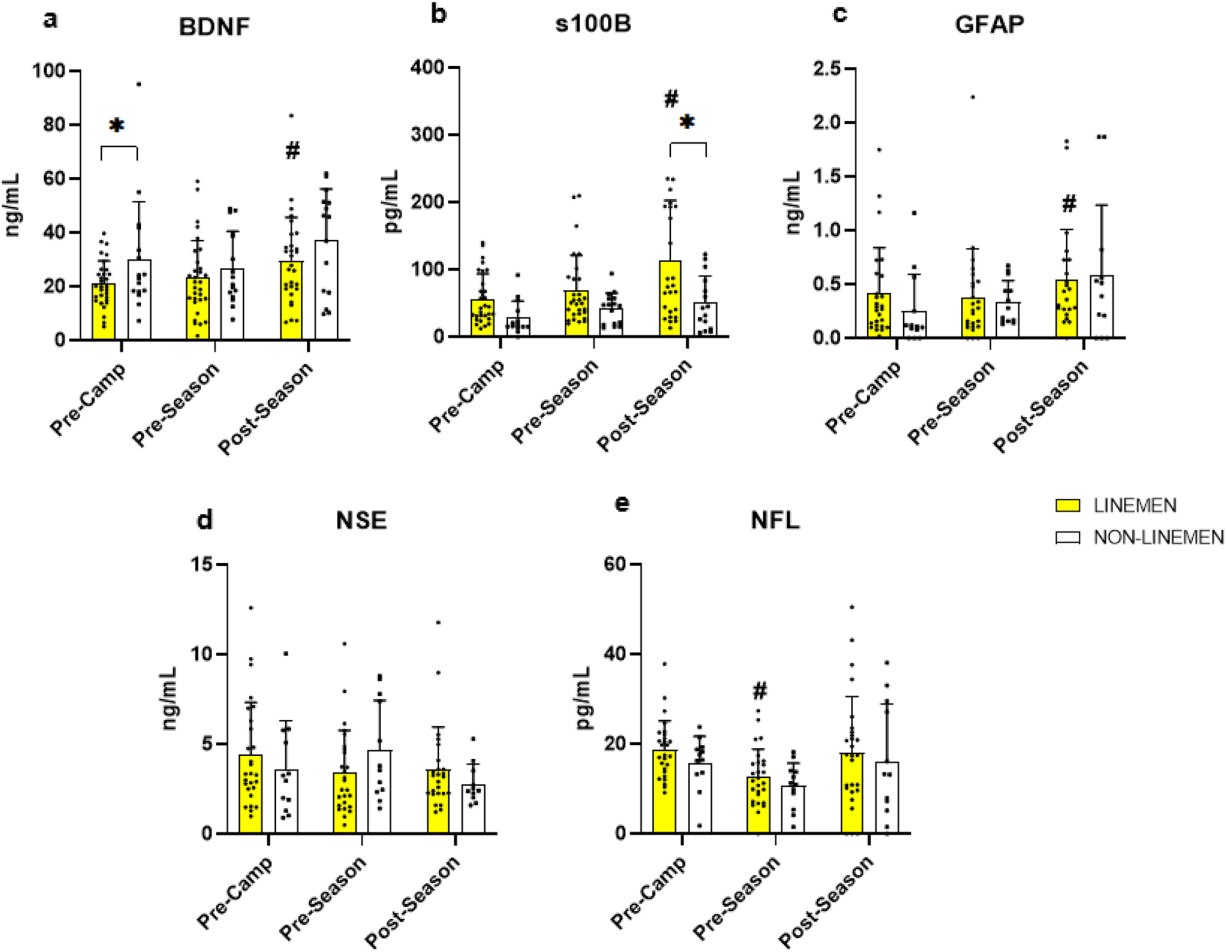
Mean ± SEM for brain-derived neurotrophic factor (BDNF), S-100 calcium-binding protein B (S100B), glial fibrillary acidic protein (GFAP), neuron specific enolase (NSE), and neurofilament light (NFL), at pre-camp, post-camp, and post-season. Hash: statistical difference from pre-camp (p < 0.05). Asterisk : statistical difference from non-linemen group (p < 0.05).

The changes in biomarkers of mTBI in linemen and non-linemen in response to pre-season training camp and a competitive season are presented in Fig. 1; Supplementary Table S1. The concentrations of circulating BDNF in linemen were lower than non-linemen at the pre-camp (linemen: 21.12 ± 8.43 ng/ml vs. non-linemen: 30.33 ± 21.23; p < 0.05); however, no differences were detected in BDNF concentrations between the groups immediately after pre-season training camp (linemen: 23.13 ± 13.93 ng/ml vs. non-linemen: 26.98 ± 13.59 ng/ml; p = 0.412) and post-season (linemen: 29.56 ± 16.17 ng/ml vs. non-linemen: 37.75 ± 20.75 ng/ml; p = 0.12). At the post-season timepoint, BDNF was higher than pre-camp in linemen (pre-camp: 21.12 ± 8.43 ng/ml vs. post-season: 29.56 ± 16.17 ng/ml; p = 0.014). Circulating S100B concentrations were not different between the groups at pre-camp (linemen: 54.98 ± 37.18 pg/ml vs. non-linemen: 28.90 ± 24.87 pg/ml; p = 0.14) and at the end of pre-season training camp (linemen: 66.90 ± 51.65 pg/ml vs. non-linemen: 43.20 ± 22.68 pg/ml; p = 0.16), however, S100B concentration was greater in linemen at the end of the season (linemen: 107.93 ± 88.96 pg/ml vs. non-linemen: 51.01 ± 39.97 pg/ml; p < 0.01), compared to non-linemen. NSE did not change throughout the study in linemen (p = 0.20), and even decreased from pre-season to post-season in non-linemen (pre-season: 4.69 ± 2.65 vs. post-season: 2.82 ± 1.07; p < 0.05). On the other hand, GFAP increased from pre-camp training to post-season in linemen, but was not different between the groups throughout the season (p = 0.46). Finally, NFL concentrations decreased during pre-season training camp (pre-camp: 19.10 ± 6.29 vs. pre-season: 12.68 ± 6.41; p < 0.01), before increasing during the competitive season (pre-season: 12.68 ± 6.41 vs. post-season: 18.42 ± 12.63; p < 0.01) in linemen, while no change was detected throughout the season in non-linemen (p = 0.16).

### Structural MRI changes throughout the season

**Figure 2:**
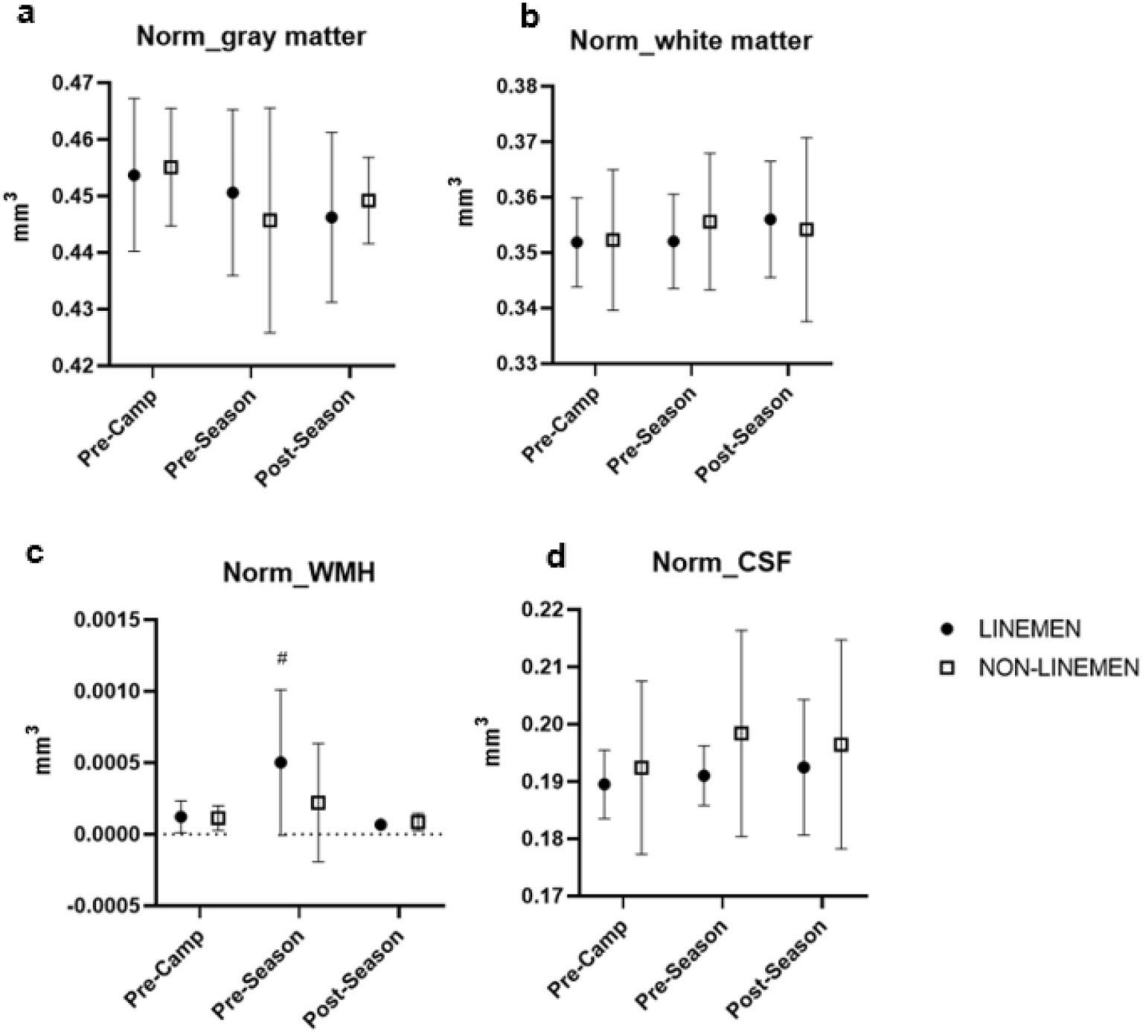
Mean ± SEM for structural MRI; normalized gray matter, white matter, white matter hypersensitivity (WMH), cerebral spinal fluid (CSF), at pre-camp, post-camp, and post-season. Hash: statistical difference from pre-camp (p < 0.05).

Changes in grey matter, white matter, cerebrospinal fluid (CSF), and white matter hyperintensities (WMH) volumes throughout the season are presented in Fig. 2 and Supplementary Table S2. Throughout the study, linemen and non-linemen presented no difference in structural MRI variables, including grey matter, white matter, and CSF (p > 0.05 for all). However, while no differences were found in white matter hyperintensities between linemen and non-linemen (p = 0.43) throughout the season, white matter hyperintensities increased in linemen during pre-season camp training (p < 0.05) before coming back to pre-camp levels at the end of the season (p < 0.01). Mean white matter fractional anisotropy (FA) and mean white matter mean diffusivity (MD) are presented in Supplementary Table S3. No changes were detected in FA and MD over the course of the season and between linemen and non-linemen (p = 0.90, p = 0.89, respectively).

### Functional MRI changes throughout the season

To further evaluate the effects of a pre-season training camp and the subsequent competitive season on brain function in linemen and non-linemen, we characterized fMRI signal changes as well as changes in response times (RT) to validated neurocognitive tasks performed during fMRI scanning, namely the Stroop and Attentional Network Test (ANT), between pre-camp and the end of pre-season training camp or the end of the season.

#### Stroop tasks

**Figure 3:**
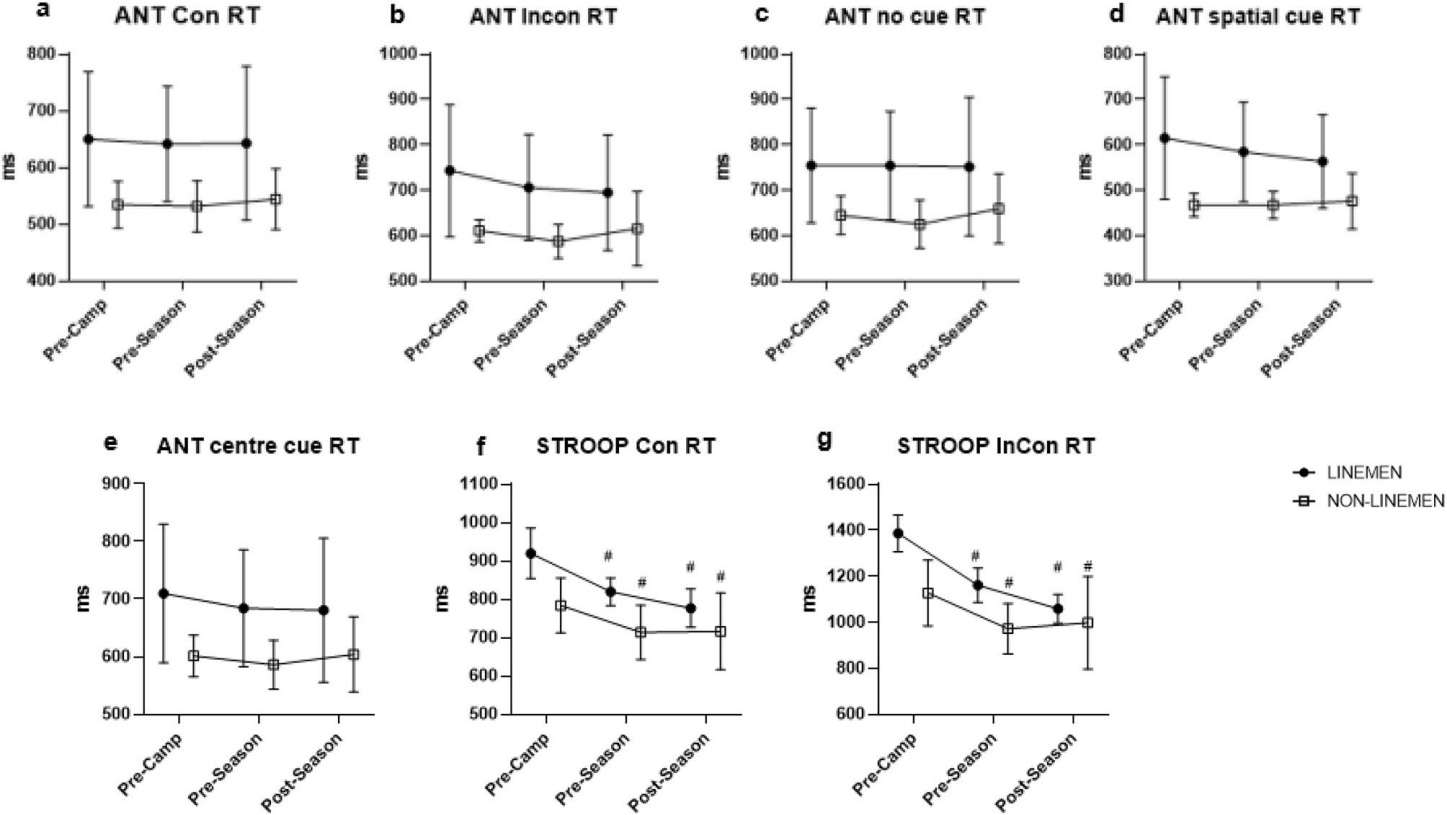
Mean ± SEM for Reaction network task) and S TROOP tasks at pre-c amp, post-camp, and post-season. Hash: statistical difference from pre-camp (p < 0.05). *Con* congruent, *InCon* incongruent.

Reaction time (RT) to congruent stimuli during the Stroop task decreased throughout the season in both linemen and non-linemen (p < 0.0001) (Fig. 3f), with no difference between the groups throughout the season (p = 0.14). Linemen showed faster responses to incongruent trials over time (pre-camp vs. post-camp training: p < 0.0001; post-camp training vs. post-season: p < 0.05), while non-linemen showed slowest RTs at pre-camp, and faster at post-camp training (p < 0.05), and neither faster nor slower at the post-season (p = 0.91) (Fig. 3g). Although the accuracy of the Stroop task was not different between the groups at pre-camp (p = 0.23), non-linemen showed higher task accuracy at the end of the pre-season training camp than linemen (p < 0.01). Interestingly, these differences in task accuracy between the two groups disappeared at the end of the season (p = 0.25). Finally, Stroop task fMRI activation in the occipital gyrus was higher in non-linemen than linemen at pre-camp (p < 0.05) but over time, non-linemen group went down, having comparable activation between linemen and non-linemen at post-season (p = 0.24).

#### Attention network test (ANT)

Changes in reaction time for the different ANT trial types over the study period are presented in Fig. 3. No differences in RT were found in response to any ANT trial type, including congruent, incongruent, no cue, center cue, and spatial cue between linemen and non-linemen throughout the study (p = 0.12, p = 0.15, p = 0.16, p = 0.17, p = 0.09, respectively). Furthermore, ANT accuracy was similar between the groups at all time points (pre-camp: p = 0.55, post-camp: p = 0.12, post-season: p = 0.24, respectively). fMRI activation to the ANT task in the right thalamus region (p < 0.01) and right superior parietal lobe (p < 0.01) increased between pre-camp and the end of pre-season training camp in linemen, and increased in the left fusiform gyrus (p < 0.01) with concomitant decreases in the left superior frontal gyrus between the end of pre-season training camp and post-season among linemen (p < 0.01), but not among non-linemen. Similarly, linemen exhibited higher activation in the left superior frontal gyrus compared to non-linemen at pre-camp (p < 0.05), but the differences disappeared at the end of pre-season camp (p = 0.08) and post-season (p = 0.75). In addition, non-linemen showed decreased activations in the left inferior frontal gyrus region from pre-camp to pre-season (p < 0.05), while no differences were detected between the groups throughout the season (p = 0.52).

### Correlation with biomarkers of mTBI and brain imaging

To further investigate the potential links between circulating biomarkers of mTBI included in this study and the brain imaging outcomes, we characterized the correlations between BDNF, S100B, NFL, NSE, and GFAP with the sMRI (i.e. normalized gray matter, normalized white matter, normalized white matter hyperintensities, normalized CSF) and fMRI (ANT, Stroop) outcomes using data aggregated from all timepoints.

**Figure 4:**
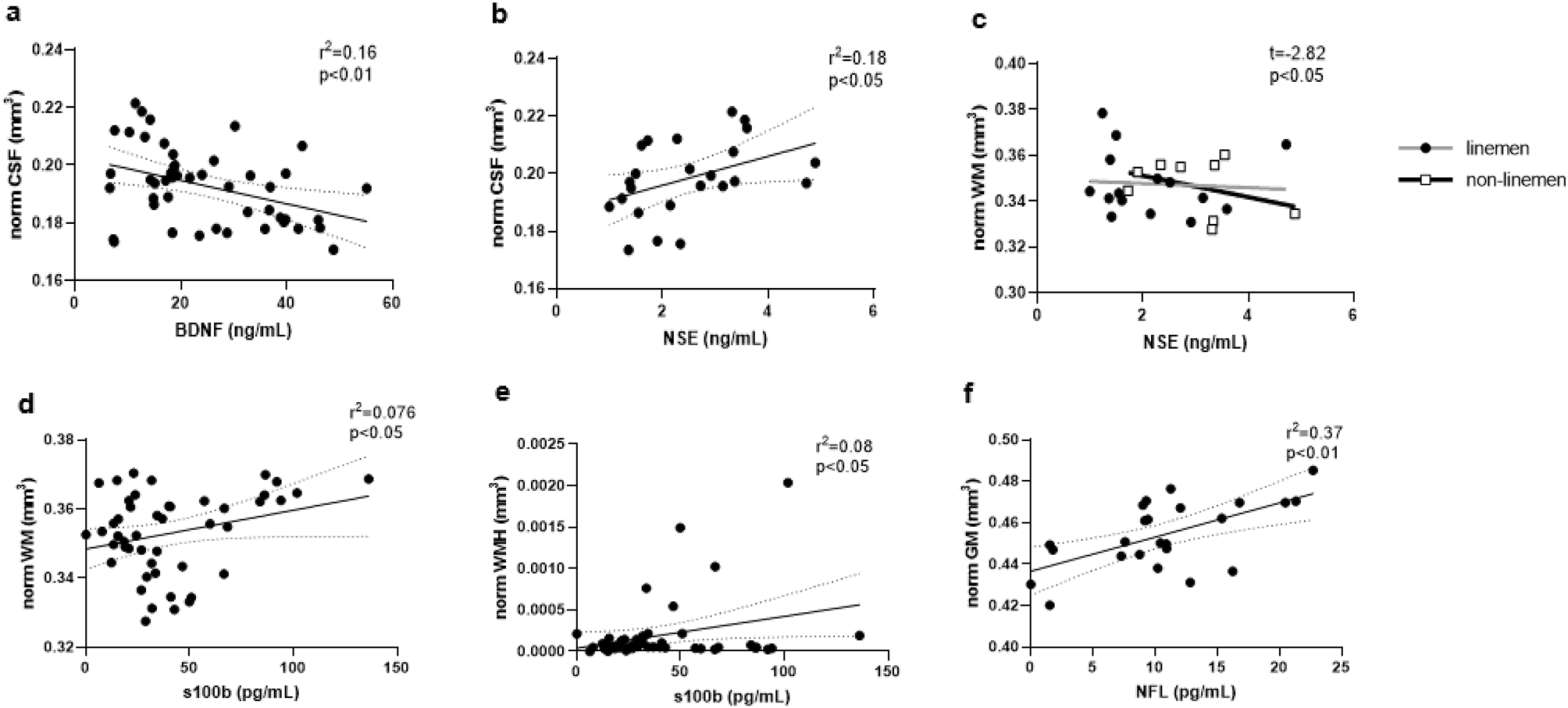
Correlations between structural MRI and biomarkers. *BDNF *brain derived neurotrophic factor, *S100B* S100 calcium binding protein B, *NSE* neuron-specific enolase, *NFL* neurofilament light chain, *CSF* cerebral spinal fluid, *WM* white matter, *WMH* white matter hypersensitivity, *GM* gray matter.

When all data were aggregated (pre-camp, pre-season training camp, and post-season, regardless of group), and accounted for repeated measures, greater circulating S100B (r^2^ = 0.076, p < 0.05) levels was associated with an increase in normalized total white matter, showing that players with higher S100B also had higher volumes of total white matter (Fig. 4). When assessing group differences between NSE and sMRI data, a higher NSE levels in linemen were associated with lesser normalized total white matter, while a higher NSE was associated with a higher volume of total white matter in non-linemen (t = −2.82, p < 0.05) (Fig. 4). On the other hand, greater circulating BDNF levels were associated with a lower normalized cerebral spinal fluid volume (CSF) (r^2^ = 0.16, p < 0.01), and a higher circulating NSE levels (r^2^ = 0.18, p < 0.05) were associated with a higher normalized CSF, regardless of group and timepoint (Fig. 4). In addition, a positive trend was observed between S100B concentrations and normalized white matter hypersensitivity (r^2^ = 0.08, p < 0.05), as well as between NFL levels and normalized total gray matter (r^2^ = 0.37, p < 0.01) in all players.

### Correlation between biomarkers and functional Magnetic resonance imaging (MRI)

No associations were found between changes in biomarker concentrations and fMRI outcomes during either camp or season in isolation (p > 0.05 for all). Consequently, data from all time points were aggregated and correlations accounting for repeated measures were calculated to increase statistical power.

#### Attention network task (ANT)

**Figure 5:**
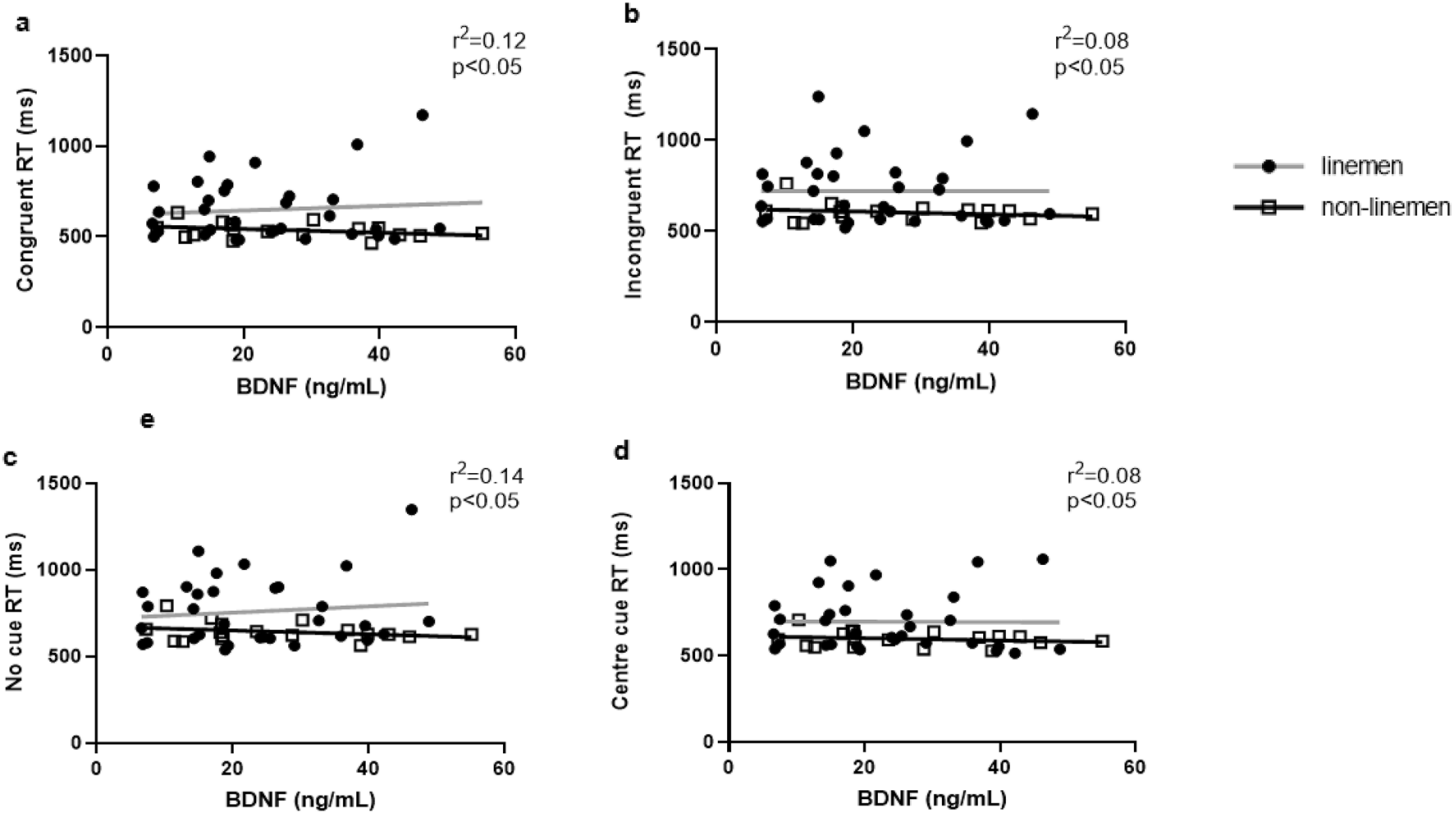
Correlations between ANT (attention network task) trials and BDNF within linemen and non-linemen players. *BDNF* brain derived neurotrophic factor, *RT* reaction time.

When analyzing data independently of timepoint, higher levels of serum BDNF were associated with slower ANT congruent RT in linemen while the opposite was true in non-linemen where higher BDNF levels were indicative of faster ANT congruent RT (r^2^ = 0.12, p < 0.05) (Fig. 5). Similarly, on ANT incongruent (r^2^ = 0.08, p < 0.05), no cue (r^2^ = 0.14, p < 0.01), and center cue tasks (r^2^ = 0.08, p < 0.05), higher BDNF levels were associated with slower RT on those tasks in linemen, while non-linemen players exhibiting higher BDNF levels also displayed faster RT on those tasks than those with lower levels of circulating BDNF (Fig. 5).

#### Stroop

**Figure 6:**
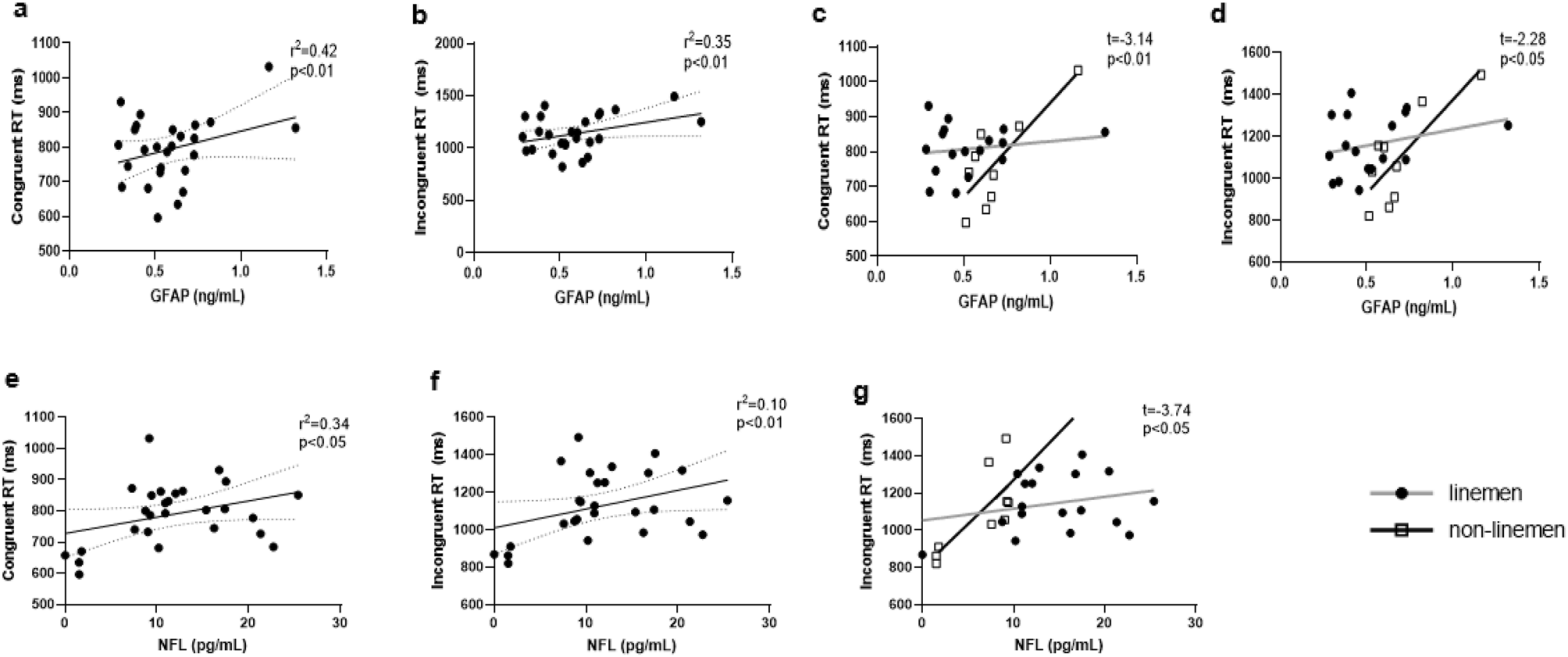
Correlations between reaction time (RT) of Stroop tasks and GFAP and NFL. *GFAP* glial fibrillary acidic protein, *NFL* neurofilament light chain.

We found greater concentrations of GFAP levels were associated with greater Stroop congruent trial RT (r^2^ = 0.42, p < 0.01), along with Stroop incongruent trials (r^2^ = 0.35, p < 0.01) (Fig. 6). In addition, greater concentrations of GFAP were observed in non-linemen who were slower on the congruent trials. Also in linemen, lower circulating GFAP was associated with slower Stroop RT (t = −3.14, p < 0.01). Similarly, while greater circulating GFAP was associated with slower RT on Stroop incongruent trials in non-linemen, low circulating GFAP was associated with slower RT in linemen (t = −2.28, p < 0.05) (Fig. 6). Moreover, a positive trend was found with linemen tending to have slower RT on Stroop incongruent tasks than non-linemen (r^2^ = 0.18, p = 0.058).

A positive correlation was found between NFL concentrations and RT on Stroop congruent tasks (r^2^ = 0.34, p < 0.05), and on RT on Stroop incongruent trial tasks (r^2^ = 0.10, p < 0.01) (Fig. 6), suggesting higher NFL concentrations were associated with slower RT on both tasks. Of note, the association between NFL levels and Stroop incongruent RT was stronger in non-linemen than linemen, with those who exhibited greater circulating NFL concentrations also demonstrating the slowest incongruent RT (t = −3.74, p < 0.05) (Fig. 6). S100b and NSE were not correlated with any of ANT or Stroop tasks (p > 0.05 for all).

## Discussion

High-impact sports such as American football are associated with repeated head collisions that vary in number, magnitude, and velocity based on players’ position. Moderate to high amplitude impacts to the head in competitive football players often lead to concussions and likely promote acute and chronic neurological impairments^1,2^. Since the rapid and accurate diagnosis of concussion can improve clinical outcomes^1,2^, numerous studies have attempted to identify blood biomarkers of mTBI in both athletes^4,8,10,11^, and trauma patients^5,9^. While some brain-derived proteins appear to be detectable in the blood of patients with severe TBI^8,10^, their sensitivity in diagnosing mTBI from non-concussive impacts in athletes who experience lower magnitude head impact is less clear. This is especially true in American football players exposed to repeated low-moderate intensity impacts to the head throughout regular training and competitive seasons. Therefore, we aimed to characterize changes in several blood biomarkers of mTBI along with their association with structural and functional MRI in elite-level collegiate football players exposed to repeated sub-concussive impacts to the head due to their position throughout the course of a season. In this study, we found that linemen exhibited greater levels of circulating S100B, GFAP, and BDNF after a competitive season compared to pre-camp season levels. Considering that no players were diagnosed with a concussion during the study period, physical activity itself may be sufficient to induce changes in circulating biomarkers of brain trauma. Indeed, some of the proteins included in this study, such as S100B, have been shown to increase in elite swimmers after a long-distance swim competition, indicating that exercise itself can elevate some of these proteins in circulation, independently of any impact to the head^12^.

Previous studies reported increases in brain-borne proteins in circulation after concussive and sub-concussive trauma, including S100B^6,11^, GFAP^6,9^, NSE^6,7,10^, NFL^6,8^, and BDNF^13^. Among these, S100B is released by astrocytes in response to brain injury and remains elevated 24–48 h following concussion^1,5,6^. GFAP is also a cytoskeletal intermediate filament protein expressed by astrocytes, highly abundant in the brain, and elevated levels of GFAP were found in mild to moderate TBI patients compared to uninjured controls^5,9^. When comparing these proteins, studies have suggested that GFAP may be more sensitive in detecting intracranial CT lesions compared to S100B^9^. Interestingly, compared to pre-camp, we showed elevations in both S100B and GFAP at the end of the competitive season. We also found a positive correlation between the levels of circulating S100B and white matter hyperintensities, a predictor of risk of stroke and cognitive impairments^13^. It is however important to highlight that white matter hypersensitivity in linemen at post-camp training was as high as those reported in non-athletic populations in other studies^14^. The lack of association between S100B and any functional neuroimaging outcomes in the present study does not enable us to draw meaningful conclusions advocating for any neurological dysfunction to have occurred in the linemen. In contrast, we also found that GFAP levels and RTs of Stroop tasks both in congruent and incongruent trials were in the intuitive direction—higher level of GFAP, worse RT which are non-invasive measurements eliciting cognitive function measuring selective attention^15^. While we were unable to adequately capture accurate data on the magnitude and number of hits to the head during games, we hypothesize that these increases in blood biomarkers could be due to repeated exposures to in-training and in-game head impacts. However, there was no association between the changes in circulating S100B and GFAP levels and the changes in mean values of sMRI or fMRI outcome. S100B and GFAP can be released in the blood in response to low-moderate impacts to the head and subsequent disruption of the blood–brain barrier, without being associated with neurological impairments, therefore limiting their diagnostical validity to identify mTBI^1,2,6^.

NSE is a promising surrogate marker for neuronal injury. Serum NSE has been shown to remain chronically elevated for up to 2 months after sport cessation in boxers who experienced repetitive head blows^7^, even in the absence of mTBI. As such, clinical validation between repetitive head trauma and elevated levels of NSE is required before asserting its usefulness as a diagnostic tool^10^. NFL, a member of the family of intermediate filament proteins, is a biomarker of axonal injury in the brain and has been reported to be elevated throughout the football season in collegiate football athletes^8^. Throughout the season, we found no changes in these biomarkers. However, greater NSE concentration was associated with the lesser normal appearing white matter in linemen while greater NSE was associated with the greater normal appearing white matter in non-linemen, which requires further investigation. One possible explanation could be that loss of white matter connections is related to the NSE concentrations. We recognize that this association may not be clinically-relevant since normal appearing white matter did not change during the season, and no associations were observed between the changes in NSE and the changes in structural MRI.

BDNF is a neurotrophic protein that plays a role in the survival, plasticity, and growth of neurons and has been shown to be increased in severe TBI compared to mild or moderate TBI^8^. However, due to the properties of BDNF, elevated BDNF concentration in the peripheral blood can have dichotomic origins. In the present study, BDNF levels were associated with ANT tasks in a group-dependent manner. Specifically, non-linemen with higher BDNF levels also exhibited faster RT on the ANT task, while linemen with higher BDNF had slower RT when performing the same task. These correlations may suggest that elevation of BDNF is associated with neural impairments due to neural damage in athletes who frequently experience low-moderate intensity impacts to the head while also being associated with improved neurological functioning in athletes who exercise but do not get hit frequently. However, we remain cautious and should not over-interpret these data without actual head impact records. Indeed, this could also be indicative of normal and healthy brain repair in response to damages induced by repeated impacts, or a natural consequence of the higher level of physical fitness in our population^16^. Especially, while S100B has been suggested to be one of the strongest biomarkers to detect head injuries, previous studies on athletes competing in non-contract sports such as swimming, running and basketball^12,17,18^ showed increased level of S100B independently of head impact history. Thus, it can be argued that none of the biomarkers included in this study are sufficient to be used as clinically-relevant diagnostic tools of mTBI.

Our study has limitations and caution should be used when interpreting the data. First, this study addressed the blood serum levels of various brain-borne proteins and their association with structural and functional MRI outcomes throughout the season in non-concussed elite-level American football athletes. However, we were unable to adequately collect head impact magnitude and numbers, thus limiting our ability to investigate the effects of a clinically-relevant range of head trauma on these outcomes as well as not including non-contact sport athletes as a control group. Due to technical constraints, a further limitation can be found in our inability to collect structural and functional MRI data on our entire study population. Finally, although this fell outside the purview of this study, future studies should aim to include concussed players in their study population.

In sum, although some studies showed that the blood biomarkers are elevated with head impact/concussion, in our study, we did not see a direct association between brain-borne proteins and sMRI and fMRI in players exposed or not to frequent head impacts. Furthermore, our study identified crucial differences in the profile of circulating biomarkers of mTBI between linemen and non-linemen over the course of a competitive season. These differences extended to brain structure, activation patterns and reaction times to the STROOP task and other functional tasks; however, these did not appear to be related to the players’ position or circulating brain-borne protein concentrations. Of note, our players were not diagnosed with mTBI with no classical symptoms of mTBI. However, the trends of biomarkers showed similar patterns of mTBI/concussed patients, thus our study advocates against the use of individual circulating brain-borne proteins as diagnostic tools for mTBI and concussion because of the lack of the sensitivity of biomarkers.

## Methods

### Participants

National Collegiate Athletic Association (NCAA) Division I football players (48 players in total: 32 linemen; 16 non-linemen) were recruited over three consecutive years in this study. All linemen and non-linemen were encouraged to enroll in the study, regardless of being on a scholarship or being a walk-on athlete. The linemen group included offensive linemen, defensive linemen, and tight ends, while the non-linemen group included defensive backs, linebackers, long snappers, punters, wide receivers, kickers, and quarterbacks. All athletes provided written informed consent, and the Institutional Review Board granted ethical approval at Louisiana State University for the study (IRB # 3902). Exclusion criteria included any conditions the team physician regarded as too risky to participate in the practices/games and any medical, psychiatric, or behavioral factors that could interfere with participating in the study. All methods were performed in accordance with the relevant guidelines and regulations.

### Study design and visits

**Figure 7:**
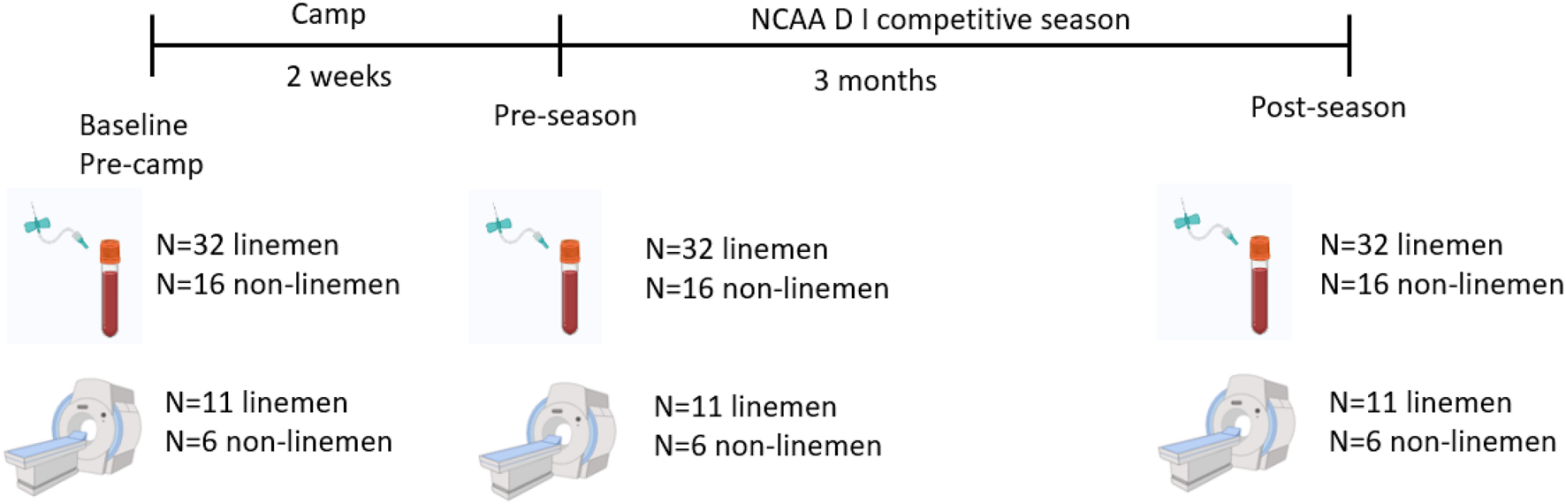
Schematic diagram of blood draw, structural MRI, and functional MRI time points (created with BioRender.com.)

The experimental protocol consisted of three study visits: (1) pre-camp, (2) pre-season, and (3) post-season (Fig. 7). These study visits occurred (1) no more than a week before the start of training camp, (2) 24 h, and 48 h for the blood collection and sMRI and fMRI respectively after the end of the 2-week pre-season training camp, (3) 24 h, and 48 h for the blood collection and sMRI and fMRI respectively after the last game of the competitive season. During the pre-camp visit, signed informed consent was collected, height (cm) and weight (kg) were measured, blood was drawn from an antecubital vein, and sMRI and fMRI scans were conducted. The blood draw and sMRI/fMRI were repeated at pre-season and post-season visits. Participants were asked to be fasted for up to 12 h before the blood draw. All blood samples were taken 24 h after practice (pre-season) or game (post-season). sMRI and fMRI were performed 24–48 h after practice (pre-season) or game (post-season) in a single season. As for MRI scanning voluntary participation was encouraged. Medical and coaching staff evaluated any player suspected of having been concussed during trainings or games, and no participant exhibited any symptom of concussion nor were they diagnosed with a concussion throughout the season.

#### Blood sampling and biomarker analysis

Fasted and resting serum samples (10 mL) were collected at the same time of the day at each timepoint using vacutainer serum separator tubes (BD, Franklin Lakes, NJ). Blood samples were centrifuged and stored at − 80 °C freezer until analyses were run in batches at the end of the study. Biomarkers of brain injury including S100 calcium-binding protein B (S100B) (MyBioSource, Inc., USA), brain-derived neurotrophic factor (BDNF) (R&D System, USA), glial fibrillary acidic protein (GFAP) (R&D System, MA, USA), neurofilament light chain protein (NFL) (MyBioSource, Inc., USA), and neuron specific enolase (NSE) (R&D System, MA, USA) were measured from serum samples using quantitative Enzyme-Linked Immunosorbent Assays (ELISA). Due to low sample volume, only 26 linemen and 13 non-linemen were included in the GFAP, NSE, and NFL analysis, while S100B and BDNF were characterized on the complete dataset of 48 participants.

#### Structural MRI, and functional MRI data collection and analysis

##### Magnetic resonance imaging (MRI)

An sMRI scan was first performed to quantify gray matter, normal-appearing white matter, cerebrospinal fluid, white matter hyperintensities, and diffusion MRI measures as indices of structural brain health. An fMRI scan was also performed during the execution of two cognitive tasks (Stroop Task and the Attention Network Task) to measure task-related changes in brain activity.

##### MRI acquisition

Brain MRI scans were performed on a GE Discovery 3 T scanner at Pennington Biomedical Research Center. A 32-channel phased array head coil was used. Sequences included: (1) T1-weighted 3D BRAVO (voxel size, 0.94 × 0.94 × 1.2 mm^3^; voxel array, 256 × 256 × 140; flip angle, 12°; NEX, 2; TI, 450; bandwidth, 31.25; total run time, 6:41); (2) axial 2D FLAIR (voxel size, 1.07 × 1.07 × 2 mm^3^; voxel array, 224 × 224 × 69; flip angle, 160°; TE, 95 ms; TR, 9000 ms; TI, 2250 ms; NEX, 1; bandwidth, 31.25; total run time, 4:58); (3) axial 2D gradient echo EPI BOLD (voxel size, 3 × 3 × 3 mm^3^; voxel array, 64 × 64 × 43; flip angle, 90°; TE, 35 ms; TR, 2500 ms; NEX, 1); (4) axial 2D single-shot spin echo EPI diffusion (30 independent gradient directions; 1 B0 image; 1.875 × 1.875 × 3.6 mm^3^ voxel size; 128 × 128 × 56 voxel array; TE: minimum; TR: 8000). Participants wore a respiratory monitoring belt and pulse oxygenation sensor to model respiratory and cardiac effects on the BOLD signal^19^.

##### fMRI tasks

First, an adaptive *Stroop Task* was administered to test inhibitory control in the context of negative feedback and time-pressured responses^20^. In each trial, for 400 to 5000 ms, participants saw one probe word and four target words that were names of colors. The task was to identify the target word whose color matched that of the probe. In the congruent (incongruent) condition, word meaning matched (did not match) the color it was printed in. Correct (incorrect) responses on three consecutive incongruent trials prompted a 300 ms reduction (increase) in stimulus duration. Four 52–60 s incongruent trial blocks were interleaved with four congruent trial blocks, each of which had the same number of trials as the previous incongruent block. The inter-block interval was 10–17 s. Stroop Task performance was summarized in terms of task accuracy (i.e., percent of trials answered correctly), mean reaction times to congruent and incongruent trials, and the so-called interference effect (i.e., the difference in mean reaction times between incongruent and congruent conditions).

Next, the *Attention Network Task (ANT)* was administered. In each trial of the ANT, a line of arrows was presented in one of two locations on the screen. Participants clicked a left-hand or right-hand button depending on whether the center arrow pointed to the left or right. The participant was required to suppress distracting flanker arrows on either side of the center arrow and had to process a spatial cue that appeared prior to the arrow line, either in the same location as the eventual arrow line, in both possible locations of the eventual arrow line, or not at all. The *alerting score* was the difference in mean reaction time (i.e.*,* the amount of time between the presentation of the line of arrows and the button click) between trials with and without a spatial cue. The *orienting score* was the difference in reaction time between trials with the spatial cue in the same vs. different location as the arrow line. The *executive control score* was the difference in mean reaction time between trials with congruent and incongruent flankers. The total number of trials was 456.

##### Structural MRI data analysis

Post-processing of structural MRI data follows previously described techniques^21–23^. Key FLAIR processing steps include manual removal of non-brain elements from the FLAIR image by operator-guided tracing of the dura mater within the cranial vault, resulting in the delineation of a total cranial volume (TCV) region; MRI non-uniformity correction of the TCV^24^; thresholding of TCV into brain and non-brain tissues^25^; fitting a single Gaussian distribution to the brain tissue intensity distribution and labeling of all voxels with intensity greater than 3.5 standard deviations above the mean as WMH^26^. Key T1-weighted image processing steps include MRI non-uniformity correction^27^; and segmentation of gray matter, white matter, and cerebrospinal fluid by a Bayesian maximum-likelihood expectation–maximization algorithm^28^.Pre-processing of diffusion MRI data followed the standardized approach taken in our earlier publications^29–31^. Briefly, eddy current correction was applied to gradient images through repeated co-registration using FSL flirt, the average gradient image was linearly co-registered to the average B_0_ image, the average B_0_ image was linearly coregistered to the corresponding T1-weighted image, and the T1-weighted image was nonlinearly deformed to a standard template space. These transformations allowed all diffusion MRI data to be placed into the standard space. Fractional anisotropy (FA) and mean diffusivity (MD) were calculated at each voxel in the native diffusion MRI space and transformed into the standard space. The primary measures of interest in subsequent analysis were volumes of total WMH, total gray matter, white matter, and cerebrospinal fluid (expressed as a percentage of TCV), and mean FA and MD across the white matter. Higher total gray matter, white matter, and mean FA, and lower WMH volume, mean MD, and cerebrospinal fluid are viewed as indicators of better brain health.

##### fMRI data preprocessing

Functional MRI data was analyzed using MATLAB R2016a and the Statistical Parametric Mapping 12 (SPM12) toolbox. Preprocessing of fMRI included slice timing correction, head motion correction, smoothing, co-registration to the T1-weighted image, and warping T1-weighted data to a standard coordinate frame. Cardiac and respiratory time series were regressed out of the data using RETROICOR^19^. Time points with excess head rotation (> 1.5°) or translation (1.5 mm) were removed from analysis. Voxel time series were entered into a first-level general linear model, where the experimental design was modeled as boxcar functions convolved with the canonical hemodynamic response function. First level beta maps, performed at the single subject level, quantified differences in BOLD signal within an individual between pairs of conditions; all such condition pairs, corresponding to our contrasts of interest, are listed in the description of tasks below. To encourage reproducibility of fMRI findings, current best practices for fMRI analysis emphasize the importance of testing effects in brain regions hypothesized a priori to be affected^32–35^. Such an approach avoids the identification of spurious fMRI effects in unexpected brain regions—a common pitfall of classical voxel-level analysis followed by application of statistically-complex multiple comparison correction methods. Therefore, BOLD signal differences were averaged within 5 mm-diameter regions of interest (ROI) that were identified before the beginning of this project as task.

##### fMRI activation analysis

The ‘inhibitory control’ contrast was measured between congruent and incongruent blocks in the Stroop Task. Data from incorrect, missing, or pre-attentive (i.e., < 200 ms) responses were removed from analysis. The 3D coordinates of Regions of Interests (ROIs) that repeatably show fMRI signal differences in this version of the Stroop Task under the inhibitory control contrast (including activated regions covering occipital, fusiform, angular, middle frontal, inferior frontal, superior frontal, cingulate, and middle temporal gyri; and deactivated regions covering the cerebellum, precuneus, insula, lentiform nucleus, and thalamus) were identified from a published report^20^. The mean beta value for the inhibitory control contrast among all voxels in a 3-mm-radius sphere centered at each ROI location was calculated. BOLD signal contrasts corresponding to the alerting, orienting, and executive control scores were assessed similarly from the ANT fMRI data at ROI locations reported previously^36^.

### Statistical analysis

All statistical analyses were performed by using JMPro 16 (SAS Inc., Cary, NC), and graphical representations were constructed using GraphPad Prism 9.00 (GraphPad Software). Serum levels of the biomarkers, sMRI, and fMRI are presented as mean ± standard deviation. Differences in the outcome measures (BDNF, S100B, GFAP, NFL, NSE, sMRI and fMRI) between the groups (linemen and non-linemen) over time (between pre-camp, pre-season and post-season) were detected using two-way repeated-measures analysis of variance (RM ANOVA). When significant differences in time/group/interaction were found between linemen and non-linemen or time, independent students’ t-tests were used to compare to determine difference. All data sets were normally distributed. For the sMRI and fMRI, a sub-sample of 17 players was included in the analyses (linemen:11, non-linemen:6). Correlations between serum biomarkers outcomes and fMRI/sMRI outcome measures were determined using Pearson’s r. Data are presented as Mean ± SD, and significance was set as p < 0.05. All figures were created using GraphPad Prism (v 9.0.0, Boston, Massachusetts, USA).

### Ethics approval and consent to participate

All participants provided written informed consent before participating, which the Louisiana State University's Institutional Review Board approved (IRB # 3902).

### Supplementary Information

Supplementary Tables.

## Data Availability

The datasets used and analyzed during the current study are available from the corresponding author on reasonable request.
