# Supplementary material for: Blood and MRI biomarkers of mild traumatic brain injury in non-concussed collegiate football players
Source: Sci Rep. 2024 Jan 5;14:665. doi: 10.1038/s41598-023-51067-3 (PMC10770029; doi:10.1038/s41598-023-51067-3)
Supplement: Supplementary file 1 — Supplementary Tables. [file 41598_2023_51067_MOESM1_ESM.docx]

Supplementary Table S1. Changes in circulating biomarkers of mTBI in linemen and non-linemen over the course of a season. # indicates difference from pre-camp baseline with significant set as p<0.05 . * indicates difference from non-linemen group with significant set as p<0.05.

|  | Linemen (n=32) | | | Non-linemen (n=16) | | |
| --- | --- | --- | --- | --- | --- | --- |
|  | Pre-camp | Post-camp | Post-season | Pre-camp | Post-camp | Post-season |
| BDNF ng/mL | 21.12±8.43* | 23.13±13.93 | 29.56±16.17 # | 30.33±21.23 | 26.98±13.59 | 37.75±20.75 |
| S100B   pg/mL | 54.98±37.18 | 66.90±51.65 | 107.93±88.96 *# | 28.90±24.87 | 43.20±22.68 | 51.01±39.97 |
| GFAP  ng/mL | 0.42±0.42 | 0.37±0.46 | 1.3±2.43# | 0.25±0.34 | 0.34±0.20 | 0.58±0.65 |
| NSE  ng/mL | 4.44±2.98 | 3.36±2.39 | 3.60±2.43 | 3.60±2.56 | 4.69±2.65 | 2.82±1.07 |
| NFL  pg/mL | 19.10±6.29 | 12.68±6.41# | 18.42±12.63 | 15.33±5.92 | 10.58±4.86 | 15.40±12.57 |

*BDNF (Brain Derived Neurotrophic Factor); S100B (S100 Calcium Binding Protein B); GFAP (Glial Fibrillary Acidic Protein); NSE (Neuron-Specific Enolase); NFL (Neurofilament Light Chain)

Supplementary Table S2. Changes in structural MRI outcome measures in linemen and non-linemen over the season. # indicates different from pre-camps baseline with significant set as p<0.05.

|  | Linemen (n=10) | | | Non-linemen (n=6) | | |
| --- | --- | --- | --- | --- | --- | --- |
|  | Pre-camp | Post-camp | Post-season | Pre-camp | Post-camp | Post-season |
| Total volume | 1556.66±161.61 | 1564.19±162.25 | 1550.07±164.23 | 1546.35±101.64 | 1558.95±102.20 | 1552.50±109.99 |
| Gray matter | 708.28±62.66 | 707.42±61.13 | 693.91±64.02 | 703.71±48.33 | 694.71±53.12 | 697.93±59.27 |
| White matter | 551.90±74.68 | 555.45±75.63 | 556.36±78.95 | 544.82±41.82 | 554.16±37.31 | 549.70±44.07 |
| WMH | 0.18±0.22 | 0.75±1.04 # | 0.11±0.09 | 0.17±0.12 | 0.36±0.65 | 0.13±0.09 |
| Total CSF | 296.22±31.18 | 300.53±32.34 | 299.65±37.17 | 297.48±29.44 | 309.59±36.57 | 304.59±30.14 |
| Total TCV | 1710.91±191.93 | 1720.07±206.73 | 1714.35±193.92 | 1600.51±128.44 | 1643.92±138.10 | 1247.91±99.31 |
| Total tissue | 1260.44±133.10 | 1263.66±131.36 | 1250.42±137.44 | 1248.87±85.76 | 1249.36±81.48 | 1637.61±135.91 |

*WMH (White Matter Hypersensitivity); CSF (cerebral spinal fluid); total cranial volume (TCV)

# Supplementary Table S3. Changes in Diffusion Tensor Imaging (DTI) outcomes measures in linemen and non-linemen over the season.

|  | Linemen (n=10) | | Non-linemen (n=6) | |
| --- | --- | --- | --- | --- |
|  | Post-camp | Post-season | Post-camp | Post-season |
| Mean white matter FA | 0.42±0.03 | 0.42±0.03 | 0.42±0.04 | 0.44±0.04 |
| Mean white matter MD  (10–3 mm2 s–1) | 0.73±0.04 | 0.70±0.03 | 0.71±0.06 | 0.74±0.04 |

*FA(Fractional Anisotropy); MD (Mean Diffusivity)
